# The feasibility and validity of the EQ-5D-Y and CHU9D in a challenging context: adolescent mental health in India

**DOI:** 10.1186/s41687-026-01028-x

**Published:** 2026-04-02

**Authors:** Samuel J. Perry, Mutharaju Arelingaiah, Sphoorthi Prabhu, Amy Palmer, Siobhan Hugh-Jones, Janardhana Navaneetham, Hareth Al-Janabi

**Affiliations:** 1 Health Economics Unit, School of Health Sciences, College of Medicine and Health, University of Birmingham, Birmingham, UK; 2 Department of Psychiatric Social Work, National Institute of Mental Health and Neuro Sciences, Bangalore, Karnataka India; 3 School of Psychology, University of Leeds, Leeds, UK

**Keywords:** Wellbeing, Psychological health, Health-related quality of life, Psychometric properties, Content validity, School-based interventions, LMICs, Cognitive interviews, Survey response theory

## Abstract

**Background:**

Outcome measurement and economic evaluation in mental health are increasingly conducted in challenging contexts, including for young people, in low- and middle-income countries, and outside of the healthcare system. This study aimed to assess the feasibility, acceptability, and content validity of two key preference-based measures (the EQ-5D-Y-3L and CHU9D) in such a context in India.

**Methodology:**

Ten cognitive interviews with semi-structured follow-up discussions and 164 surveys were conducted with adolescents in Indian schools. Coding of the cognitive interviews and semi-structured follow-up discussions was conducted to identify completion challenges. The surveys contained both instruments and open-ended follow-up questions, and were analysed to further explore the feasibility, acceptability, and content validity of the instruments.

**Results:**

Error rates were higher for the EQ-5D-Y-3L than for the CHU9D in the cognitive interviews, principally due to the ‘Having pain or discomfort’ item, and difficulty in reporting what occurred ‘today’. A lower proportion of adolescents completed the full EQ-5D-Y-3L instrument in the surveys. The CHU9D was the more preferred instrument by participants for reasons including having more relevant content, particularly regarding mental and psychological health, and more response options. However, some participants indicated neither instrument covered all important aspects of their quality-of-life, including social interactions, education, and material circumstances.

**Conclusions:**

Researchers might prioritise the CHU9D as an instrument for measuring quality-of-life in school-going Indian adolescents. However, the measure was not without limitations, and careful attention should be paid to understanding age- and country-specific factors that pose challenges to its usage in economic evaluations.

**Supplementary Information:**

The online version contains supplementary material available at 10.1186/s41687-026-01028-x.

## Background

Economic evaluations are conducted to provide evidence on the cost-effectiveness of healthcare interventions, and, increasingly, in contexts outside of the healthcare system such as schools to provide evidence of value-for-money. It is in the recommended guidelines for many countries to present the results of these economic evaluations using quality-adjusted life years (QALYs) [1]. Calculating QALYs for economic evaluation typically relies on patient-reported outcome measures (PROMs) which capture generic health-related quality of life (HRQoL) [2]. Given the influence that the results of economic evaluations can have on policy, it is imperative that the PROMs utilised to elicit HRQoL are reliable, valid, and responsive to change in the population where they are administered [3]. However, applying these instruments to children and adolescents presents methodological and practical challenges, particularly in low- and middle-income countries (LMICs) [4]. Existing work has either adapted adult instruments such as the EQ-5D-Y-3L [5], or developed new child-friendly instruments such as the CHU9D [6]. However, the use and assessment of psychometric properties of such instruments has primarily been in upper-middle- and high-income countries [7]. 

Assessing QoL change from preventive interventions in school-based settings also poses additional challenges such as ceiling effects, and generic QoL instruments may overlook important aspects of preventive intervention outcomes [8]. These challenges could be more pronounced in adolescents from LMICs, where different domains of QoL may be emphasised [9]. Such concerns are not unfounded, with studies suggesting that instruments like the EQ-5D-Y-3L and CHU9D may be prone to ceiling effects [10–14]. 

India has the world’s largest adolescent population [15]. Approximately 7.3% of Indian adolescents experience clinical mental health conditions [16], and investment in school-based mental health and wellbeing interventions has been advocated [17, 18]. Addressing this, the Safeguarding Adolescent Mental Health in India (SAMA) Project has codesigned an evidence-based, whole school, multi-component intervention to address risks and protective factors for adolescent anxiety and depression to promote wellbeing in Indian adolescents [19]. However, there is a lack of evidence on the use of generic QoL instruments to assess the benefits of such an intervention. Moreover, there is a paucity of studies in LMICs measuring and valuing childhood health utilities, with many relying on cruder measures such as visual analogue scales to derive health utility scores [20]. 

To our knowledge, neither the EQ-5D-Y-3L nor the CHU9D have been used in India. Other instruments like the Paediatric Quality of Life Inventory 4.0 (PedsQL) and the World Health Organization Quality of Life-Brief (WHOQOL-BREF) have been applied to Indian adolescents [21, 22], however they lack the ability to produce value sets from which to derive health utility scores reflecting the preferences of specific populations. Hence, the objective of this study was to evaluate the feasibility, acceptability, and content validity of the EQ-5D-Y-3L and CHU9D for use with Indian school-going adolescents. To this end, we conducted 10 cognitive interviews and administered 164 surveys to adolescents in SAMA-participating schools.

## Methods

### Overview

The EQ-5D-Y-3L is a child-friendly version of the adult EQ-5D generic measure of health status [5]. It is most appropriate for use in children and adolescents aged 8 to 15 years and is available in more than 100 languages including English and Kannada (the official language of the Indian state of Karnataka where SAMA was delivered). The measure comprises a descriptive system assessing health in five dimensions (Table 1). These are similar to the adult version, bar some small changes to the wording and meaning of some items and answer options to enhance comprehension amongst children [5]. Each dimension is self-assessed on three increasing severity levels.

**Table 1:** EQ-5D-Y-3L – instrument details

Dimension	Severity Levels	Reference Point
Mobility (walking about)	I have no problems… (1) I have some problems… (2) I have a lot of problems… (3)	Today
Looking after myself	I have no problems… (1) I have some problems… (2) I have a lot of problems… (3)	Today
Doing usual activities	I have no problems… (1) I have some problems… (2) I have a lot of problems… (3)	Today
Having pain or discomfort	I have no problems… (1) I have some problems… (2) I have a lot of problems… (3)	Today
Feeling worried, sad, or unhappy	I am not… (1) I am a bit… (2) I am very… (3)	Today

**Notes**: The EQ-5D-Y-3L was available from EuroQol in both English and Kannada

The CHU9D is an alternative measure of children’s QoL, originally developed for children aged 7 to 11 years but since validated for use with adolescents up to 17 years [6, 23]. Its descriptive system consists of nine dimensions (Table 2) assessed on five increasing levels. Originally produced in English, it has been translated into multiple languages, however, no Kannada translation is available. A Kannada translation was produced by colleagues who are native speakers of Kannada and fluent in English (MA, SP) from the National Institute of Mental Health and Neurosciences India (NIMHANS), independently performing and then internally validating this using forward-back translation. This was presented in a tabulated format with text in both English and Kannada, and for reasons of space contained only the dimensions and not the additional examples provided for ‘Daily routine’, ‘Schoolwork/homework’, and ‘Able to join in Activities’ in the original CHU9D.

**Table 2:** CHU9D – instrument details

Dimension	Severity Levels	Reference Point
Worried	I don’t feel… (1) I feel a little… (2) I feel a bit… (3) I feel quite… (4) I feel very… (5)	Today
Sad	I don’t feel… (1) I feel a little… (2) I feel a bit… (3) I feel quite… (4) I feel very… (5)	Today
Annoyed	I don’t feel… (1) I feel a little… (2) I feel a bit… (3) I feel quite… (4) I feel very… (5)	Today
Tired	I don’t feel… (1) I feel a little… (2) I feel a bit… (3) I feel quite… (4) I feel very… (5)	Today
Pain	I don’t have any… (1) I have a little bit of… (2) I have a bit of… (3) I have quite a lot of… (4) I have a lot of… (5)	Today
Sleep	I had no problems… (1) I had a few problems… (2) I had some problems… (3) I had many problems… (4) I couldn’t sleep at all (5)	Last night
Daily routine	I have no problems with… (1) I have a few problems with… (2) I have some problems with… (3) I have many problems with… (4) I can’t do… (5)	Today
Schoolwork/Homework	I have no problems with… (1) I have a few problems with… (2) I have some problems with… (3) I have many problems with… (4) I can’t do… (5)	Today
Able to join in activities	I can join in with any… (1) I can join in with most… (2) I can join in with some… (3) I can join in with a few… (4) I can join in with no… (5)	Today

**Notes**: The CHU9D was available in English. An in-house Kannada translation was performed by NIMHANS researchers fluent in both English and Kannada

Cognitive interviews and surveys were administered to evaluate the feasibility, acceptability, and content validity of the EQ-5D-Y-3L and CHU9D when used with school-going Indian adolescents. Content validity, defined as the degree to which the content of an instrument adequately reflects the construct to be measured, was assessed in accordance with the COSMIN guidelines [24]. This outlines three key considerations of the relevance, comprehensiveness, and comprehensibility of an instrument. Relevance entails whether the items included are relevant for the construct of interest, the target population of interest, and the context of use of interest, alongside whether the response options and recall period are appropriate. Comprehensiveness determines whether there are no key concepts missing, whilst comprehensibility includes understanding of instructions, items, and response options by the population of interest as intended, alongside whether the items are appropriately worded and the response options match the question [24]. Face validity can be considered as an additional component of validity, reflecting whether the items are sensible and appropriate for the given population [25]. Assessment of face validity involves ascertaining the comprehensiveness and acceptability of an instrument. When comparing multiple instruments, acceptability can be reflected by users’ preferences between measures [26]. Feasibility reflects the ease of completion of an instrument, thereby assessing its comprehensibility by ascertaining whether the measure is usable in the relevant context [26]. 

### Study participants and data collection

This study was conducted as part of the SAMA codesign and feasibility study, which recruited eight schools in the Indian state of Karnataka (specifically, semi-urban Bengaluru and rural Kolar). Participants in this phase of the SAMA study were Grade 9 pupils (typically aged 14 to 15 years old). Five schools were randomised as intervention schools and three were randomised as waitlist schools to receive the interventions 3 months later [19]. For the present study, participants were recruited from three of the intervention schools for the cognitive interviews and the three waitlist schools for the surveys. Further details of the school recruitment process into SAMA are available full study protocol [19]. 

#### Cognitive interviews

Cognitive interviews were conducted to explore adolescents’ response processes as they completed the outcome measure questionnaires. The think-aloud approach was primarily used, in which participants were encouraged to verbalise their thought processes while answering each item [27]. This open-ended approach allows interviewers to ascertain how respondents interpret questions and any problems they encounter, while also reducing the possibility of interviewer-imposed bias. As such, this in-depth investigation of the participants’ response process enables the identification of any issues which threaten the content validity of the measures in question, such as the comprehension of the items as intended or the comprehensibility of the instructions [24, 29]. 

The think-aloud approach to cognitive interviews has been used in previous studies to assess the feasibility and content validity of other PROMs with various populations, including carers, patients, and older adults [26, 28–31], as well as in preference elicitation studies using discrete choice experiments [32]. An alternative approach to cognitive interviewing is verbal probing, whereby the interviewer follows up after a participant’s response to a question to explore their reasoning behind their answer or other relevant information related to the question [27]. Importantly, these approaches are not necessarily mutually exclusive, and in practice both can be combined flexibly within a cognitive interview when this supports participants articulating their reasoning [27]. 

For the cognitive interviews, following full parental and adolescent informed consent, ten adolescents were convenience sampled from two semi-urban government schools in Bengaluru and one rural private school in Kolar. Eligibility required participants to be able to communicate in English or Kannada.

The interviews were conducted in schools in January 2023 by two male researchers – SJP (MSc., Research Fellow in Health Economics at the University of Birmingham) and MA (PhD, Research Manager in the Department of Psychiatric Social Work at NIMHANS). SJP is a native speaker of English, whilst MA is a native speaker of Kannada and fluent in English. The interviewers had not previously met with the participants, although participants had some prior knowledge of SAMA. For five of the interviews conducted in a single-sex girls’ school, the SAMA lay counsellor at the school was present in the room alongside the adolescent at their request, however the lay counsellor had no input into the interview process. For the other five interviews the adolescent was alone. In the first five interviews, participants received the EQ-5D-Y-3L in English and the CHU9D in English with Kannada translations beneath (but were encouraged to read in English). This was done to ascertain any differences in the participants’ response process when presented with the instruments in English or Kannada. In the subsequent five interviews, participants received the official Kannada translation of the EQ-5D-Y-3L and the same English-Kannada bilingual format of the CHU9D (but were encouraged to read in Kannada).

The interviews began with the interviewers introducing themselves to the participant and providing a brief explanation of the purpose of the research, alongside a reminder to the adolescents that they were free to withdraw from the interview and study at any time. This was followed by a bilingual explanation of the tasks and a ‘window counting’ warm-up exercise to induce thinking aloud [27]. Participants were provided with paper copies of the EQ-5D-Y-3L and CHU9D, and were asked to complete them at their own pace while thinking aloud. Participants were encouraged to think-aloud in Kannada, with MA providing real-time translations in English to SJP to ensure he followed the interview process and facilitate questioning in the follow-up discussion. For adolescents who struggled to think aloud, interviewers primarily adopted a verbal probing approach, prompting participants to read each item aloud, select their response, and then explain their choice.

A follow-up discussion was guided by stem questions (Table S1 in supplementary materials). These questions were designed to further assess the validity of the measures, in accordance with COSMIN guidelines [24], by ascertaining if each measure was comprehensive, relevant, and acceptable for the adolescent users, clarify the feasibility of completion, and elicit preferences between the two measures.

Interviews typically took between 15 and 25 min, and were audio recorded and transcribed verbatim. Any segments of the interview in Kannada were first transcribed in Kannada and then translated to English by MA, with internal checks conducted by an additional team member who is a native speaker of Kannada and fluent in English (SP). Transcription of the English segments of the interview was conducted and consolidated by both MA and SJP.

#### Survey

A paper-based survey was administered to further assess the content validity of the EQ-5D-Y-3L and CHU9D. Participants were recruited from a private school and a government school in Bengaluru, and a government school in Kolar. Surveys were administered to all adolescents for whom parent consent and adolescent assent had been obtained.

The paper-based survey included the EQ-5D-Y-3L, CHU9D, and six free text questions similar to those posed in the follow-up discussion of the cognitive interviews to provide additional data on the feasibility, acceptability, and content validity of the instruments in accordance with COSMIN guidelines (Table S2 in supplementary materials) [24]. The survey was available in both English and Kannada, with respondents encouraged to answer in their preferred language. The survey was administered by the NIMHANS research team in school classrooms one to four weeks prior to the SAMA intervention commencing in the schools (between January and March 2023).

### Data analysis

#### Interview analysis

To assess feasibility of completion, the portions of each interview where the participants answered the EQ-5D-Y-3L and CHU9D were divided into 14 ‘segments’, with each segment reflecting a dimension on the instruments. As such, there were five for each EQ-5D-Y-3L dimension and nine for each CHU9D dimension. Thus, a total of fifty segments pertaining to the EQ-5D-Y-3L and ninety pertaining to the CHU9D were analysed across the ten interviews. Three independent raters (SJP, AP, HA) analysed each segment, identifying problems that participants faced when completing the instruments in relation to translation, comprehension, retrieval, judgement, and response [33]. Each segment was coded as error-free, containing one or more of the five error types, or as a ‘struggle’ indicating that respondents faced difficulties arriving at their response without displaying defined error types (Table S3 in supplementary materials) [30]. Any discrepancies in coding between the raters were resolved through discussion.

Thematic analysis was applied to the entire interview transcripts using a coding framework developed to further assess aspects of content validity [24, 34]. The framework included five codes: comprehensiveness, response options, clarity, format, and preference. Sub-codes were developed to identify themes within these codes. Coding was conducted in NVivo 12.

#### Survey analysis

Quantitative and qualitative analyses were conducted on the survey responses to further evaluate the feasibility, acceptability, and content validity of the instruments. The proportion of missing responses for each instrument was calculated to assess feasibility of completion, with responses considered missing if they were left blank or were otherwise invalid. Descriptive statistics were produced reflecting the frequency of response choice and mean score for each item. To assess ceiling effects, the proportion of respondents choosing response level one on all items was calculated for each instrument. The six follow-up questions were analysed using the thematic framework established from the interviews, and the results from these analyses were combined. Kannada-to-English translations for the follow-up questions were performed by SP.

## Results

### Sample characteristics

Cognitive interviews were conducted with Grade 9 pupils (14 to 15 years old) including seven girls (five from a rural private school and two from a semi-urban government schools) and three boys (from a semi-urban government school). No additional information was collected on the participants.

Responses from 164 Grade 9 adolescents were received for the survey. Respondents attended three schools: a semi-urban government school (47%) in Bengaluru, a rural government school in Kolar (37%), and a semi-urban private school in Bengaluru (16%). Approximately 49% of respondents were female. Only students from the private school chose to answer the survey in English. Participants ranged from 12 to 17 years old, with a mean age of 14.5. Note that this range in ages reflects that some students may have either been held back in school due to poor academic performance, admitted late in early schooling years, or previously dropped out between school years due to family issues or migration.

### Cognitive interview error scoring

Table 3 provides details of errors which occurred on both instruments with supporting quotes. Participants were able to answer most items on the EQ-5D-Y-3L, but all exhibited at least one error or a struggle. Thirteen (26%) of the fifty segments were associated with at least one error or struggle. These issues were exhibited exclusively on the items ‘Having pain or discomfort’ and ‘Feeling worried, sad, or unhappy’. For the former, four of the five participants who received the Kannada EQ-5D-Y-3L did not understand the Kannada translation for ‘discomfort’ (‘asaukhya’). Three participants made comprehension errors as they interpreted pain or discomfort as psychological not physical, which contravenes the intended definition of pain and discomfort of “physical or bodily hurt, but not psychological or mental suffering” [5]. Three participants made retrieval errors by answering the item generally rather than reflecting on their feelings ‘today’. Similar retrieval errors were made by five participants on the item ‘Feeling worried, sad, or unhappy’, whilst one participant made a response error as they said they were “Not worried, sad, or unhappy” whilst answering the EQ-5D-Y-3L but subsequently stated they were “A bit worried” when answering ‘Worried’ on the CHU9D.

**Table 3:** Examples of Errors/Struggles on the EQ-5D-Y and CHU9D

Error Type	EQ-5D-Y		CHU9D	
	Dimension (*N* Errors)	Example Quote	Dimension (*N* Errors)	Example Quote
Translation	POD (4)	*“I don’t have pain sir*,* but what is ‘asaukhya’?” – AB3 (Translation Error – Having pain or discomfort)*		
Comprehension	POD (3)	*“I have some pain or discomfort. My mother won’t be there at home*,* I feel sad for that… If there are issues with friends*,* then I feel sad.’ – AG7 (Comprehension Error – Having pain or discomfort)*	DRT (4) SWK (1) PAI (2)	*“I don’t feel any pain. I feel happy and worried for you have come today” – AG1 (Comprehension Error – Pain)*
Retrieval	POD (3) WSU (5)	*“I am a bit worried*,* sad*,* or unhappy. If my friends don’t speak to me then I feel unhappy” – AG4 (Retrieval Error – Feeling worried*,* sad*,* or unhappy).*	DRT (1) SWK (1) ACT (1)	*“I have a few problems in my daily routine*,* when teachers scold us” – AG2 (Comprehension and Retrieval Error – Daily routine)*
Judgement			SWK (1) ACT (3)	*“I have no problems with my schoolwork/homework today. If I want to be good in the future*,* then I should listen to parents and teachers” – AG3 (Retrieval and Judgement Error – Schoolwork/Homework)*
Response	WSU (1)	*“I feel a bit worried today” [on CHU9D item ‘Worried’] – AG3 (Response Error – Feeling worried*,* sad*,* or unhappy)*	DRT (1)	*“I have no problems with my daily routine. I do all of my activities sir*,* but today I didn’t do since I was having some pain” – AG1 (Response Error – Daily routine)*
Struggle			PAI (1) TIR (1) DRT (1) SWK (1)	*“Tired means rest*,* isn’t it?” – AB1 (Struggle – Tired)*

EQ-5D-Y: ‘POD’ is ‘Having pain or discomfort’, ‘WSU’ is ‘Feeling worried sad, or unhappy’. CHU9D: ‘ACT’ is ‘Able to join in activities’, ‘DRT’ is ‘Daily routine’, ‘PAI’ is ‘Pain’, ‘SWK’ is ‘Schoolwork/Homework’, ‘TIR’ is ‘Tired’. Full supporting data with the quotations used to determine the number of errors is available on request

One participant completed the CHU9D without error. Seventeen (19%) of the ninety segments were associated with at least one error or a struggle. Four items were answered by all participants without issue: ‘Worried’, ‘Sad’, ‘Annoyed’, and ‘Sleep’. The item with the highest error/struggle rate was ‘Daily routine’, with six (60%) participants exhibiting errors/struggles. Four comprehension errors were made as the participants failed to understand what the item was asking them, referring to factors such as having tests or being scolded by teachers as causing them issues in their daily routine. One participant exhibited a struggle, as although he understood the item, he questioned his ability to answer it as his day had not yet been completed. This participant also questioned the wording of the item ‘Schoolwork/Homework’ in the same manner. Two participants also exhibited errors on this item. ‘Able to Join in Activities’ had the second highest error/struggle rate (40%), largely driven by judgement errors where participants reasoned their response through the quantity of activities they were doing rather than their ability to do them. Two comprehension errors and a struggle were exhibited on the item ‘Pain’, with two participants interpreting pain as psychological, and one questioning whether the item solely meant physical pain. As with the EQ-5D-Y-3L, this contradicts the intended meaning of the item reflecting physical pain or hurt [6]. Finally, one respondent struggled with ‘Tired’ as he indicated he was unsure what was meant by the Kannada words provided for tired (‘ayasa’ or ‘danivu’). He was prompted by MA to consider an alternative Kannada word, ‘sustu’, which is used more colloquially in everyday speech to convey a feeling of tiredness/weariness. The participant then stated he understood this term better than the words ‘ayasa/danivu’, which are used in more formal written language, and answered the question without error. 

### EQ-5D-Y-3L and CHU9D survey completion

Table 4 provides the response level distributions and mean response scores for the five EQ-5D-Y-3L items across the surveys. Response level one was most frequently chosen for all items, while 59 (36%) of respondents chose level one for all five items. The completion rate was 92%, with 3% of items missing or invalid. For the CHU9D (Table 5) the modal response level was level one for seven items, with level two the modal response for the items ‘Worried’ and ‘Tired’. Response level one was chosen for all items by 28 participants (17%). The completion rate was 95%, with 1% of items missing or invalid.

**Table 4:** EQ-5D-Y response level descriptive statistics

Item	L1 %	L2 %	L3 %	Miss %	Mean
MOB	78	17	2	3	1
LAM	90	5	2	2	1
DUA	85	13	1	2	1
POD	67	24	6	2	1
WSU	54	34	7	5	2
**Total**	75	19	4	3	1

**Notes**: *N* = 164. Figures correct to nearest whole number. ‘MOB’ is ‘Mobility’, ‘LAM’ is ‘Looking after myself’, ‘DUA’ is ‘Doing usual activities’, ‘POD’ is ‘Having pain or discomfort’, WSU is ‘Feeling worried, sad, or unhappy’. L is level. ‘Miss’ is missing or invalid

**Table 5:** CHU9D response level descriptive statistics

Item	L1 %	L2 %	L3 %	L4 %	L5 %	Miss %		Mean
WOR	37	45	11	2	4	1	2	
SAD	49	32	9	3	6	1	2	
PAI	49	32	10	1	7	1	2	
TIR	37	39	13	3	6	2	2	
ANY	58	30	7	3	1	1	2	
SWK	59	31	5	3	2	1	2	
SLP	70	13	4	2	8	3	2	
DRT	65	26	6	1	2	1	1	
ACT	64	16	12	6	2	1	2	
**Total**	54	29	8	3	4	1	2	

Notes: *N* = 164. Figures correct to nearest whole number. ‘WOR’ is ‘Worried, ‘SAD’ is ‘Sad’, ‘PAI’ is ‘Pain’, ‘TIR’ is ‘Tired’, ‘ANY’ is ‘Annoyed’, ‘SWK’ is ‘Schoolwork/Homework’, ‘SLP’ is ‘Sleep’, ‘DRT’ is ‘Daily routine’, ‘ACT’ is ‘Able to join in activities’. ‘L’ is level. ‘Miss’ is missing or invalid

### Instrument properties identified through interviews and surveys

Below we present the themes relating to the feasibility, acceptability, and content validity of the EQ-5D-Y-3L and CHU9D, first relating to the specific issues for each instrument (Table 6), then to the generic issues across the two instruments (Table 7).

**Table 6:** Instrument-specific properties

Properties	EQ-5D-Y	CHU9D
Clarity		
Translation and Language	Translation for Discomfort: *“Discomfort (‘asaukhya’) is also required*,* but translation sir”– AB3 (Interview)* Meaning of Discomfort: *“If we are doing something*,* if that is not feeling good for me*,* then it is [discomfort]” – AG6 (Interview)* Translating EQ-5D-Y to Kannada: *“Bit of difficulty understanding… Yes sir*,* it would be” [when asked if the EQ-5D-Y had been in Kannada] – AG2 (Interview)*	Phrasing of Items: *“Sir*,* what do you mean whole day is not it? I mean*,* a day isn’t it? Here they have asked today*,* today is not completed? Without day completion how can I answer this sir? I can’t answer this [Daily Routine]… This [Schoolwork/Homework] is also asked like the same as above sir” – AB3 (Interview)*
**Preference**		
Content	*“The first measure as it summed up all the daily problems in a simple and accurate way” – R8 (Survey)*	*“I prefer measure two because it can access our mental health better. It gives good insight on our overall routine and emotions” – R2 (Survey)*
Options	*“This one sir… few options” – AG2 (Interview)*	*“This one sir. There are many options here in this questionnaire. People can choose different options” – AG3 (Interview)*

**Table 7:** Generic instrument properties

Properties	Exemplar quote(s)
Comprehensiveness	
Missing concepts	*“I personally feel that such measures just capture a part of health and quality of life. They probably capture 75% of the whole aspect. I feel that irritants and any argument-like aspects can alter everyday mood.” – R27 (Survey)*
Influences on response	*“Attitude and situations at family sir… there is financial difficulty at home*,* we are moving*,* like this… If you would have asked “How is your economical condition?” then I would answer either good*,* bad*,* very bad – like that” – AG7 (Interview)*
**Response Options**	
Sufficiency	*“Yes*,* the number of options were perfect because there were more than one way to express the answer. Example*,* some*,* few.” – R11 (Survey)* *“I feel enough options were there sir*,* if you would have asked us like “How are you feeling? What is your opinion?” then I think I would be able to answer even better sir” – AG7 (Interview)*
**Clarity**	
Ease of Completion	*“I would have filled it easily sir… How I did it now*,* that’s how I would have done it” – AB2 (Interview)* *“Sitting here and doing is easier sir… understanding of some of the meaning*,* confusing words… you were here to help me” – AG3 (Interview)*
Translation and Language	*“In one place the physical and in the other the psychological component [of pain] can be retained” – AG7 (Interview)*

#### Instrument-specific properties

Specific clarity issues revolved around translation and language. As highlighted in the cognitive interview error scoring, there was difficulty with the Kannada translation for ‘discomfort’ on the EQ-5D-Y-3L. Even on the English EQ-5D-Y-3L, there was confusion with the intended meaning of the word, with one respondent in the interview outlining that discomfort to her meant not “feeling good”. Some participants who received the English EQ-5D-Y-3L indicated they would have found the instrument easier to complete in Kannada. For the CHU9D, the primary misunderstanding during the interviews resulted from the items ‘Daily Routine’ and ‘Schoolwork/Homework’. One participant believed he could not answer them as his day had not been completed, whilst several participants misunderstood the intended meaning of the item, perhaps as the CHU9D did not include the examples from the official format. One survey participant also indicated they did not understand the item ‘Able to Join in Activities’, which also lacked the examples given in the official CHU9D.

Across both interviews (80%) and surveys (65% of those expressing a preference), the CHU9D was the more preferred instrument. Participants often indicated they found the CHU9D more comprehensive in covering their day-to-day routine and their mental and emotional health. However, some stated they preferred the content of the EQ-5D-Y-3L as it summed up their daily problems in a more simple and accurate way or related more to their wellbeing. Having more response options was also cited by some participants as a reason for preferring the CHU9D as this allowed them to choose a response more suited to how they were feeling. However, having fewer response options was given as a reason by a small number of individuals who preferred the EQ-5D-Y-3L as they found it easier to complete.

#### Generic instrument properties

Findings that were general to both instruments related to comprehensiveness, response options, and clarity. Exemplar quotes from the follow-up discussions (interviews) or questions (survey) pertaining to these themes are provided in Table 7, with respondents talking generally about both instruments.

Comprehensiveness was divided into ‘missing concepts’ and ‘influences on responses’. Across the interviews and surveys, participants generally felt the instruments encompassed most of the aspects important to their QoL. However, seven interviewees and nine survey respondents felt that the instruments overlooked significant ‘internal’ and ‘external’ factors. Internal factors encompassed emotions like happiness, anger, psychological distress, emotional stability, and mindset. External factors included school, education, food/hunger, social interactions, and home circumstances.

Many of the reported missing external factors had a noticeable influence on participants’ responses during the interviews. Participants often mentioned experiences such as reprimands from teachers and academic pressures. Home life was also an important influence, covering issues such as parental pressures and material circumstances. Some participants considered their adequate food consumption or lack of hunger as positively contributing to their health and wellbeing while responding to questions on both questionnaires. Social interactions with friends and engagement in sports and hobbies were also frequently cited. For internal influences, some respondents mentioned suffering from a cold when answering multiple items, while pain was considered by three respondents while responding to the items ‘Sleep’, ‘Daily Routine’, and ‘Mobility’.

We questioned participants in both interviews and surveys regarding the adequacy of response options. Most participants indicated they found the number of options sufficient. When directly asked, most respondents did not express a clear preference between the three options on the EQ-5D-Y-3L and the five options on the CHU9D. One interviewee indicated a preference for an open-ended question format to provide more precise responses. A similar sentiment was echoed by a survey respondent.

We divided clarity into sub-codes of ‘Ease of completion’ and ‘Translation and language’. Given many interviewees sought confirmation that they were doing the questionnaire correctly, to assess ease of completion we asked if they felt they would have been able to complete the instruments at home by themselves. Some indicated they would have found it easy, but others stated they would have had some difficulty. The survey asked about the clarity of the instructions, with 134 (87% of non-missing responses) reporting the instructions were clearly explained. Several survey participants noted that they found the instruments easy to answer because the content was relevant to their lives.

Regarding translation and language, the interviews highlighted a significant issue related to the interpretation of ‘pain’ on both instruments as psychological rather than physical. One respondent suggested that the instruments should contain separate items to distinguish between physical and psychological pain. Participants also expressed a preference for the instruments being in Kannada, with some interviewees who received the English versions noting they would have found it easier in their own language. Furthermore, most survey respondents opted to answer the Kannada versions of the instruments. 

## Discussion

### Key findings

Our findings indicate that compared to the EQ-5D-Y-3L, the CHU9D was more feasible for use with school-going Indian adolescents, with a lower error rate in the cognitive interview analysis (19% versus 26%) and a higher survey completion rate (95% versus 92%). This completion rate for the EQ-5D-Y-3L was below that observed in a study of five European countries and South Africa (98% to 100%) [35]. The CHU9D also had a higher acceptability than the EQ-5D-Y-3L, with it being the most preferred instrument across respondents in the interviews and survey. The CHU9D also exhibited greater content validity, with most respondents believing it was more relevant and comprehensive as an assessment of their QoL.

Error rates on both instruments were higher than those typically found in cognitive interviews using adult QoL instruments and samples [28, 30]. This was likely due to the population of school-age Indian adolescents in this study. First, adolescents might be expected to have higher error rates than adults due to their stage of cognitive development, whilst error rates would also be expected to be higher given both instruments were developed in a Western context and therefore the concepts and items may resonate less well in a different cultural context.

One of the primary sources of errors on the EQ-5D-Y-3L were temporal retrieval errors whereby participants answered items generally rather than expressing their feelings ‘today’. This issue has been found by other studies using the adult version of the EQ-5D [28]. Fewer retrieval errors were made on the CHU9D, suggesting that the reiteration of the word ‘today’ on each item’s response levels may have reinforced respondents to answer based on their present feelings. However, one participant questioned the appropriateness of using ‘today’ as a recall period for two items on the CHU9D, suggesting that he could not answer as his day had only just begun.

Most participants who received the Kannada version of EQ-5D-Y-3L struggled to understand the Kannada word for discomfort, ‘asaukhya’, likely due to it being a literal translation not commonly used in Kannada. To improve comprehensibility, an alternative translation of ‘aswastate’ (ಅಸ್ವಸ್ಥತೆ) could be utilised. This translates literally as disorder but conveys the intended meaning of discomfort.

An error common to both instruments was the interpretation of ‘pain’ as psychological rather than physical as intended. This may have been driven by participants generally being in good physical health alongside mental health being on their minds given their participation in SAMA. Further, a key theme emerging from both the surveys and interviews was the need for both instruments to include more items related to mental health, and respondents may have regarded the pain-related items as an opportunity to express their mental or emotional health. Thus, specifying physical pain may enhance the comprehensibility of both instruments.

For the CHU9D, errors were most prevalent among the interview participants on the items ‘Daily Routine’ ‘Schoolwork/Homework’ and ‘Able to Join in Activities’. The CHU9D administered lacked the examples provided in the official format for these items, which may have reduced comprehension. Despite this, the CHU9D was still more feasible than the EQ-5D-Y-3L, suggesting its superiority as an instrument for school-going Indian adolescents.

Preferences for the CHU9D could mainly be attributed to its content and greater response options. Participants felt it better captured important aspects of their daily lives, including mental and emotional wellbeing, and the five response options allowed for more tailored responses. This was evidenced by the CHU9D being less prone to ceiling effects, with 17% of participants reporting full health compared to 36% for the EQ-5D-Y-3L. This aligns with findings from studies in other populations [10, 11]. It should be noted that a five-level EQ-5D-Y version is in development (but not yet officially available), which may reduce this disparity in preference. Nevertheless, it remains that many participants preferred the content of the CHU9D.

While the CHU9D showed better content validity in terms of relevance, comprehensibility, and comprehensiveness, some adolescents felt that both instruments fell short in capturing important influences on their health and quality of life. External factors, such as school and home life, had a significant impact on responses and were identified as areas where both instruments could have provided better coverage. It is not surprising that the school environment was important to our respondents given the substantial time they spend there. Home life also exerted a significant influence on respondents, with many mentioning parental pressures to study and issues with material circumstances. Indeed, an item capturing economic conditions was suggested by one interview participant to be included by the instruments. Finally, participants believed that emotional and mental health aspects could be better captured by both instruments, although the CHU9D was seen as superior in this regard as it delineated emotions like worry, sadness, and included other emotional aspects such as annoyance. This insufficiency in the capturing of psychosocial aspects of health is not entirely surprising and has been raised with the adult version of the EQ-5D [36]. 

### Limitations and further research

Findings should be considered in light of study limitations. First, the cognitive interviews deviated from the intended think-aloud method. Many participants were understandably nervous, necessitating the introduction of verbal probing to the cognitive interviews with participants reading each item aloud, selecting their answer, and then being prompted to explain their choice. In addition, some participants sought clarification when encountering unfamiliar terms (e.g., the Kannada translation for ‘discomfort’). In these cases, interviewers provided neutral clarifications to enable participants to continue verbalising their thought process. This support may have reduced response errors (e.g., should the participant have failed to provide any response if the term had not been clarified). However, by enabling participants to proceed and continue articulating their reasoning, this support may have also increased the number of errors we observed in other aspects of the response process.

Second, the CHU9D was presented in a tabulated format with English and Kannada, which impacted its readability, and was also missing some examples. Furthermore, although the Kannada version was produced and validated by two native speakers of Kannada who are fluent in English, this process varies from that performed by EuroQol for the translation of the EQ-5D-Y-3L. That said, no translation errors were identified on the CHU9D during the cognitive interviews. Third, our sample was restricted to one geographical area of India, and thus the findings may not necessarily be replicated elsewhere given the large and diverse nature of the country.

Overall, our findings suggest that whilst the CHU9D may be the more promising instrument for use in this context, adaptations such as distinguishing between physical and psychological pain may be helpful. More research is needed on the degree to which content and language of generic QoL measures should be context specific. Further research may also assess other psychometric properties of both instruments in the context of Indian adolescents, such as reliability. Furthermore, there are currently no tariffs available for the calculation of health utilities from the value sets produced by either instrument reflecting the preferences of Indian adolescents (although work on valuing the adult EQ-5D is currently on-going) [37]. 

## Conclusion

In conclusion, in this context of school-going adolescents in the state of Karnataka, the CHU9D was more feasible, acceptable, and had better overall content validity than the EQ-5D-Y-3L. This suggests researchers might prioritise the CHU9D as an instrument for measuring adolescent QoL for economic evaluation in India. However, this does not mean the instrument is perfect, and careful attention ought to be paid to understanding country- and age-specific factors that may pose challenges to its usage, as well as providing careful supporting information about its intended application.

## Supplementary Information

Below is the link to the electronic supplementary material.

Supplementary Material 1

Supplementary Material 2

## Data Availability

The data that support the findings of this study will be made available on at least one of the UK Data Archive, MRC Researcher Data Gateway, or ReShare.

## Abbreviations

LMICs: Low- and middle-income countries
NIMHANS: National Institute of Mental Health and Neurosciences India
SAMA: Safeguarding Adolescent Mental Health in India
QoL: Quality of Life
WHOQOL-BREF: World Health Organisation Quality of Life-Brief
