# Supplementary material for: The feasibility and validity of the EQ-5D-Y and CHU9D in a challenging context: adolescent mental health in India
Source: J Patient Rep Outcomes. 2026 Apr 2;10:79. doi: 10.1186/s41687-026-01028-x (PMC13172131; doi:10.1186/s41687-026-01028-x)
Supplement: Supplementary file 1 — Supplementary Material 1 [file 41687_2026_1028_MOESM1_ESM.docx]

**Supplementary Materials**

**Table S1. Interview Stem Questions**

| **Question** | **Properties Assessed** |
| --- | --- |
| Were there any things you felt were missing or should be added to the questionnaires?  *(Prompt: Were there any areas of your life affected by your health that you thought were missing?)* | Comprehensiveness, Relevance |
| Was any wording difficult to understand? Or was any of it not very relevant or meaningful to you? | Comprehensibility, Feasibility |
| How clear did you find the instructions for the questionnaires?  *(Prompt: Do you think you could have completed them at home by yourself without any help?)* | Comprehensibility, Feasibility |
| Did you find the number of options for answering the questions OK?  *(Prompt: Would you have preferred more or fewer?)* | Relevance |
| Did you have any (other) difficulties with answering the questions? For example… *link any challenges observed during the interview here.* | Comprehensibility, Feasibility |
| Which of the two measures did you prefer and why?  *(Prompt: Which do you feel better reflects how your life is at the moment and why?)* | Acceptability |

Table S1 provides details of the six stem questions which were used to guide the semi-structured follow-up discussion after the adolescents had completed both instruments. The adolescents were asked to think about both questionnaires, with additional probing of any instrument-specific issues conducted if evident during the think-aloud task.

**Table S2. Survey Follow-up Questions**

| **Question** | **Properties Assessed** |
| --- | --- |
| **Q1:** Do you think the two measures capture what is important to your health and quality of life? For example, is there anything you would add or take away from the measures? | Comprehensiveness, Relevance |
| **Q2:** How clear did you find the instructions for the two measures? Were there any aspects that were confusing and/or could be better explained? | Comprehensibility, Feasibility |
| **Q3:** Was any wording difficult to understand on either of the two measures? | Comprehensibility, Feasibility |
| **Q4:** Did you find the number of options available for answering each question OK? For example, would you have preferred more or fewer options? | Relevance |
| **Q5:** Did you have any (other) difficulties in answering the two measures? | Comprehensibility, Feasibility |
| **Q6:** Which of the two measures did you prefer? Why? | Acceptability |
| **Notes:** Each question was provided in Kannada with the English translation below. | |

**Table S3. Error Type Definitions**

| **Error Type** | **Definition** |
| --- | --- |
| Translation | A translated word not conveying the same meaning as in the original language. |
| Comprehension | Misunderstanding the item in the way the researcher intended. |
| Retrieval | Unsuccessfully recalling the relevant information to answer the item from long-term memory. |
| Judgement | Incorrectly judging how recalled information should be used to answer the question. |
| Response | Failing to format the recalled information to provide a valid response to the questionnaire. |
| Struggle | Not displaying any of the defined error types but experiencing some other form of difficulty before reaching an appropriate answer. |
